# The Cytotoxicity of *Cotyledon orbiculata* Aqueous Extract and the Biogenic Silver Nanoparticles Derived from the Extract

**DOI:** 10.3390/cimb45120631

**Published:** 2023-12-14

**Authors:** Caroline Tyavambiza, Mervin Meyer, Adedoja Dorcas Wusu, Abram Madiehe, Samantha Meyer

**Affiliations:** 1 Department of Science and Innovation–Mintek Nanotechnology Innovation Centre, Department of Biotechnology, University of the Western Cape, Cape Town 7530, South Africa; carolinetyavambiza@gmail.com (C.T.); memeyer@uwc.ac.za (M.M.); awusu@uwc.ac.za (A.D.W.); amadiehe@uwc.ac.za (A.M.); 2 Department of Biomedical Sciences, Cape Peninsula University of Technology, Cape Town 7535, South Africa; 3 Nanobiotechnology Research Group, Department of Biotechnology, Faculty of Natural Sciences, University of the Western Cape, Cape Town 7530, South Africa

**Keywords:** green nanotechnology, silver nanoparticles, *Cotyledon orbiculata*, cell toxicity, in vitro assays

## Abstract

Green synthesized silver nanoparticles (AgNPs) have become popular because of their promising biological activities. However, for most of these nanoparticles, the cytotoxic effects have not been determined and their safety is not guaranteed. In a previous study, we successfully synthesized AgNPs (*Cotyledon*-AgNPs) using an extract of *Cotyledon orbiculata*, a medicinal plant traditionally used in South Africa to treat skin conditions. *Cotyledon*-AgNPs were shown to have significant antimicrobial and wound-healing activities. Fibroblast cells treated with extracts of *C. orbiculata* and *Cotyledon*-AgNPs demonstrated an enhanced growth rate, which is essential in wound healing. These nanoparticles therefore have promising wound-healing activities. However, the cytotoxicity of these nanoparticles is not known. In this study, the toxic effects of *C. orbiculata* extract and *Cotyledon*-AgNPs on the non-cancerous skin fibroblast (KMST-6) were determined using in vitro assays to assess oxidative stress and cell death. Both the *C. orbiculata* extract and the *Cotyledon*-AgNPs did not show any significant cytotoxic effects in these assays. Gene expression analysis was also used to assess the cytotoxic effects of *Cotyledon*-AgNPs at a molecular level. Of the eighty-four molecular toxicity genes analysed, only eight (*FASN*, *SREBF1*, *CPT2*, *ASB1*, *HSPA1B*, *ABCC2*, *CASP9*, and *MKI67*) were differentially expressed. These genes are mainly involved in fatty acid and mitochondrial energy metabolism. The results support the finding that *Cotyledon*-AgNPs have low cytotoxicity at the concentrations tested. The upregulation of genes such as *FASN*, *SERBF1*, and *MKI-67* also support previous findings that *Cotyledon-*AgNPs can promote wound healing via cell growth and proliferation. It can therefore be concluded that *Cotyledon*-AgNPs are not toxic to skin fibroblast cells at the concentration that promotes wound healing. These nanoparticles could possibly be safely used for wound healing.

## 1. Introduction

Silver has a long history of use in the medical field. Its use has been recorded from as early as 4000 B.C.E by the ancient Greeks, Romans, and Egyptians [1,2]. It was used to make different silver utensils such as plates, cups, and containers [3]. It was believed that the use of silver utensils could preserve food and water and could also prevent people from getting infectious diseases [1,3]. After silver was recognized to have antimicrobial activity, it was also incorporated into many other aspects of medicine. It was used for the treatment of infected wounds, burn wounds, skin ulcerations, and to prevent gonococcal ophthalmic infections in new-born babies [4]. Silver has also been used as a coating for medical catheters (urinary, venous, drainage catheters), surgical blades, and needles in order to prevent bacterial growth on the surfaces of these implants and medical instruments [5,6]. Due to technological advancements and the emergence of nanotechnology, the synthesis of silver nanoparticles has become popular. Silver nanoparticles are known to exert greater antimicrobial activities than silver metal because of their smaller size and higher surface area [3,7]. They were reported to be effective against drug-resistant microorganisms such as methicillin-resistant *Staphylococcus aureus* (MRSA) [8,9] and *Pseudomonas aeruginosa* [8]. Because of their properties, AgNPs are also applied in food preservation and packaging materials, water treatment, cosmetics, clothing and textiles, biosensing, and imaging [10].

Prolonged use of high doses of silver is, however, not recommended. The prolonged use of silver on skin has been associated with agyria, a condition in which silver deposition in normal skin and tissues causes discoloration. However, chronic argyria does not cause any physiological or pathologic alterations; it is only cosmetically undesirable [1,3,11]. The toxicity of AgNPs has also been reported. Nanoparticles exert their toxicity through reactive oxygen species (ROS) generation, mitochondrial dysfunction, membrane damage, and protein oxidation. Their toxicity is determined by their physicochemical characteristics which include shape, size, surface coating, and concentration [12,13]. Chemically synthesized nanoparticles have been associated with toxicity mostly because of the way they are synthesized. The chemical synthesis of AgNPs involves the use of toxic chemicals such as sodium borohydride and sodium citrate [14,15]. To reduce the toxicity of nanomaterials, researchers and scientists have turned to green nanotechnology, a field in which nanomaterials are synthesized using biomaterials obtained from plants and microorganisms instead of hazardous inorganic chemicals. Phytochemicals present in plant extracts act as both reducing and capping agents in nanoparticle synthesis [15]. Even though green synthesized nanoparticles are expected to be safer than the chemical synthesized ones, some studies state that toxicity can be attributed to the silver ions released from the AgNPs [11,13,16], meaning that green nanoparticles may still be toxic. It is therefore important to determine the toxicity of the synthesized green nanoparticles before their application.

*C. orbiculata*, a medicinal plant indigenous to South Africa, was successfully used to synthesize AgNPs (*Cotyledon*-AgNPs) [17]. *Cotyledon*-AgNPs have a size of 40–60 nm and exhibit good antimicrobial, anti-inflammatory [17], and wound-healing properties [18]. Their antimicrobial activity was comparable to, and in some instances better than, the activity of commercial antimicrobial drugs, ampicillin and fluconazole [17]. The *C. orbiculata* plant is used in traditional medicine to treat wounds, boils, and acne [19]. In our previous study, we demonstrated that *C. orbiculata* extract and *Cotyledon*-AgNPs increased the growth of HaCaT (keratinocyte cell line), KMST-6 (fibroblast cell line), and CHO (epithelial cell line) cells at concentrations of 15 and 2.5 µg/mL, respectively. At these concentrations, the wound-healing scratch assay showed that the scratch gap closed faster in the treated cells compared with the untreated control [18]. Gene expression studies using a wound-healing gene panel showed that the *Cotyledon*-AgNPs and the *C. orbiculata* extracts promoted keratinocyte and fibroblast proliferation and migration by upregulating genes such as *FGF7* and *FGF10*. They also upregulated several genes (*COL5A3*, *COL14A1*, *ITGB1*, *ITGB6*, *ACTA1*, and *TAGLN*) involved in collagen construction, extracellular matrix formation, cell adhesion, and cytoskeleton organization [18]. *Cotyledon*-AgNPs can thus be used as potential wound-healing agents. It is therefore also important to evaluate their potential cytotoxic effects at the concentrations at which these AgNPs show wound-healing activities. Thus, the aim of this study was to evaluate the toxicity of *Cotyledon*-AgNPs in KMST-6 cells at these concentrations. This was achieved by determining the effects of the *Cotyledon*-AgNPs on oxidative stress and apoptosis in KMST-6 cells. Considering the limitations of bioassays in studying the effects of nanomaterials, this study also used gene expression analysis to assess the effects of the *Cotyledon*-AgNPs on the expression levels of genes involved in toxicity.

## 2. Materials and Methods

### 2.1. Cell Culture

KMST-6, HaCaT, and CHO cells were obtained from the DSI/Mintek NIC laboratory at the University of the Western Cape (Cape Town, South Africa). The KMST-6 and HaCaT cells were grown in Dulbecco’s modified eagle medium (DMEM) supplemented with 10% fetal bovine serum (FBS) and 1% Pen-strep. CHO cells were grown in Hams-F12 media supplemented with 10% fetal bovine serum (FBS) and 1% Pen-Strep. The cells were maintained in a humidified atmosphere of 5% CO_2_ in a 37 °C incubator (SL SHEL LAB, Sheldon manufacturing, Cornelius, OR, USA).

### 2.2. Determination of IC_50_

The IC_50_ values of the *C. orbiculata* extract and *Cotyledon*-AgNPs were determined using the WST1 assay (Sigma-Aldrich, St. Louis, MO, USA). Briefly, cells were seeded in 96 well plates (1 × 10^4^ cell/well) and incubated for 24 h. After incubation, the cells were exposed to different concentrations of the *C. orbiculata* extract and *Cotyledon*-AgNPs for 24 h. The treatments were replaced with 10% WST-1 reagent diluted in appropriate culture medium. After a 3 h incubation, the absorbance was measured at 440 nm (reference 630 nm) using a microplate reader (POLARstar Omega plate reader, BMG-Labtech, Ortenberg, Germany). The IC_50_ values were calculated using the GraphPad Prism 6 software.

### 2.3. ROS Assay

The levels of ROS were determined by flow cytometry using the cell permeable fluorogenic dye CM-H_2_DCFDA. This dye diffuses into cells and is deacetylated to a non-fluorescent compound by intracellular esterases; it is then oxidized by ROS into a highly fluorescent compound dichlorofluorescein (DCF), which can be detected using the flow cytometer. The resulting fluorescence intensity will therefore be proportional to the levels of ROS within the cell. The assay was performed according to a method by [20] with modifications. In brief, KMST-6 cells were seeded in 24 well plates at a density of 1 × 10^5^ cells/mL, at standard culture conditions (5% CO_2_ at 37 °C). After 24 h, the cells were treated with *Cotyledon*-AgNPs, *C. orbiculata* extract, and 0.5% hydrogen peroxide (positive control); the negative control cells were left untreated. All the cells were incubated for a further 24 h at standard conditions. After incubation, the cells were trypsinized, washed with phosphate-buffered saline (PBS) and incubated with 200 µL of diluted CM-H_2_DCFDA (7.5 µM) for 30 min at 37 °C in the dark. Following incubation, the CM-H_2_DCFDA solution was removed, and the cells were washed with PBS. Then, 200 µL of fresh media was added to the cells and the fluorescence readings were immediately read on a BD Accuri C6 flow cytometer (BD Biosciences, San Jose, CA, USA).

### 2.4. APOPercentage^TM^ Assay 

The apoptotic effects of the *Cotyledon*-AgNPs were determined using the APOPercentage™ assay (Biocolor Ltd., Carrickfergus, Ireland) following the method by Meyer et al. [21]. Briefly, KMST-6 cells were seeded in 12 well plates and incubated for 24 h at 37 °C. The cells were treated with *Cotyledon*-AgNPs, *C. orbiculata* extract and the positive control (hydrogen peroxide). The cells were incubated for a further 24 h, trypsinized, centrifuged and stained with 250 µL of the APOPercentage™ dye. After a 30 min incubation with the dye, the stained cells were washed, centrifuged, and resuspended in 300 µL of 1× PBS. Analysis was performed using the BD Accuri C6 flow cytometer.

### 2.5. Gene Expression Studies Using the Human Molecular Toxicology PathwayFinder RT2 Profiler PCR Array

Gene expression studies were conducted according to the method used by [22]. Briefly, KMST-6 cells were seeded in 25 cm^2^ cell culture flasks at 2 × 10^5^ cell/mL. The cells were treated with *Cotyledon*-AgNPs, untreated flasks were used as controls. All experiments were performed in triplate. After a 24 h incubation, the cells were trypsinized, centrifuged and collected in 2 mL Eppendorf tubes. Total RNA extraction was performed using the RNeasy Mini Kit (Qiagen, Germantown, MD, USA) according to the manufacturer’s instructions. The RNA concentration and integrity were determined using a Qubit^®^ 2.0 Fluorometer (Invitrogen by Life Technologies, Carlsbad, CA, USA) and 1% agarose gel electrophoresis respectively. After checking the RNA quality, cDNA synthesis was achieved using the RT2 First Strand Kit (Qiagen, Germantown, MD, USA). The synthesized cDNA was used for RT-qPCR, which was performed using the Human Molecular Toxicology PathwayFinder RT2 Profiler PCR Array (Qiagen, Germantown, MD, USA). The RT-qPCR assay was conducted on the Roche LightCycler 480 (Roche, Basel, Switzerland). Data collected from the LightCycler 480 were analyzed using the Qiagen GeneGlobe Data Analysis Center (https://geneglobe.qiagen.com/za/ (accessed on 23 November 2022)). The cycle threshold (CT) values were used to determine fold changes. Genes with a fold change of ≥±1.5 and *p*-values of <0.05 (comparing the treated samples with the untreated control samples) were considered as differentially expressed genes and were used for further analysis. The Database for Annotation, Visualization and Integrated Discovery (DAVID; version 6.7) and Search Tool for the Retrieval of Interaction Genes/Proteins (STRING; https://string-db.org/ (accessed on 25 November 2022)) pathways were used to further analyze the different interactions of the differentially expressed genes.

## 3. Results and Discussion

### 3.1. Synthesis of Cotyledon-AgNPs

The *Cotyledon*-AgNPs were successfully synthesized and characterized using UV-Vis, dynamic light scattering (DLS), and high resolution transmission electron microscopy (HR-TEM) in our previous study [17].

### 3.2. The Cytotoxicity of C. orbiculata Extracts and Cotyledon-AgNPs

To assess the cytotoxic effects of the *C. orbiculata* extract and *Cotyledon*-AgNPs, their toxicity was evaluated in KMST-6, HaCaT, and CHO cell cultures using the Water-Soluble Tetrazolium 1 (WST-1) assay and the results were used to determine their half maximal inhibitory concentration (IC_50_) values. The IC_50_ values represent the concentration of a compound or treatment that inhibits the viability of cells by 50% [23]. The *C. orbiculata* extract had higher IC_50_ values compared with the *Cotyledon*-AgNPs and is therefore less toxic than the *Cotyledon*-AgNPs synthesized from it. The level of toxicity of the *Cotyledon*-AgNPs varied significantly between the cell lines, as shown by the different IC_50_ values in KMST-6, HaCaT, and CHO cells (Table 1). Table 1 also shows that the *Cotyledon*-AgNPs were more toxic to the HaCaT and CHO cells than KMST-6 cells. A previous study showed that the *C. orbiculata* extract and *Cotyledon*-AgNPs promoted wound healing at 15 and 2.5 µg/mL, respectively [18]. These concentrations were also several-fold lower than the IC_50_ values determined for the *C. orbiculata* extract and *Cotyledon*-AgNPs. The fibroblast cell line, KMST-6, was selected in this study to further evaluate the toxic effects of *C. orbiculata* extract and *Cotyledon*-AgNPs since this cell line was also used in a previous study to demonstrate the wound-healing effects of *C. orbiculata* extract and *Cotyledon*-AgNPs [18].

### 3.3. Effects of C. orbiculata Extracts and Cotyledon-AgNPs on Cellular ROS Levels

Metallic nanoparticles have been reported to increase ROS levels inside cells leading to oxidative stress and toxicity. Actually, ROS production is said to be the most common mechanism of cellular toxicity by nanoparticles [12]. ROS are reactive chemicals that contain oxygen [24]. These by-products of oxygen metabolism include hydrogen peroxide, superoxide anion radicals, and hydroxyl radicals. Under normal conditions, ROS play an important role in various cellular signaling pathways including growth regulation [25]; however, unusually high levels of ROS may have detrimental effects on the cells. Excessive ROS can damage the cellular antioxidant defense systems by increasing oxidative stress while reducing the amounts of glutathione and superoxide dismutase enzymes [26]. ROS generation also affects redox homeostasis causing lipid peroxidation and protein carbonylation. This leads to the damage of DNA, proteins, and lipids, eventually causing apoptosis [12]. Nanoparticles have been shown to increase ROS levels by disrupting the electron transfer process, disturbing mitochondrial function, and interfering with the expression of genes involved in oxidative stress [24,27]. The toxicity of AgNPs has been attributed to the particle itself and the Ag ionic species that may be released from the nanoparticle [26,28,29,30].

**Figure 1:**
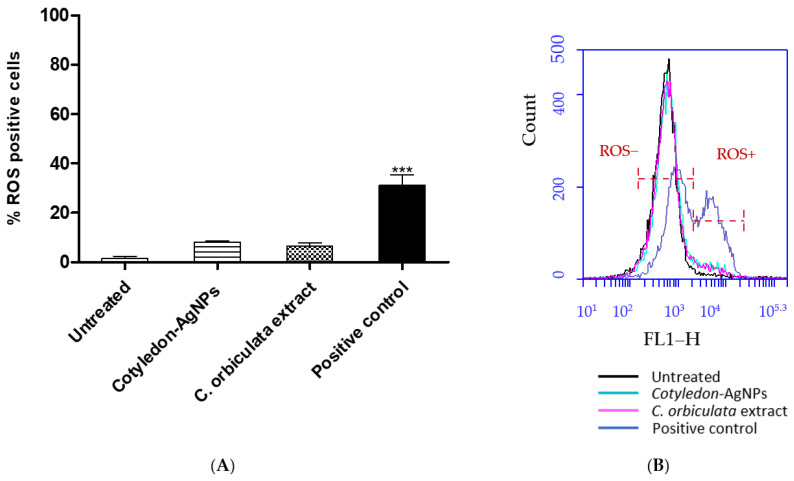
(**A**) shows the effects of *Cotyledon*-AgNPs (2.5 µg/mL) and *C. orbiculata* extract (15 µg/mL) on ROS levels in KMST-6 cells. (**B**) shows an example of a histogram plot of CM-H_2_DCFDA probe fluorescence that was generated by flow cytometry for KMST-6 cells. Indicated in the histogram are populations that are positive (ROS+) and negative for (ROS−) for CM-H_2_DCFDA fluorescence. Each value represents mean ± standard error of the mean (SEM, n = 3); statistical significance of the *C. orbiculata* extract- and *Cotyledon*-AgNPs-treated cells when compared with the untreated cells is indicated with *** for *p* <0.001.

The 5-(and-6)-chloromethyl-2′,7′-dichlorodihydrofluorescein diacetate, acetyl ester (CM-H_2_DCFDA) oxidative stress probe was used to determine the effects of *C. orbiculata* extract and *Cotyledon*-AgNPs on ROS production in the fibroblast cells (KMST-6). Neither the *C. orbiculata* extract or the *Cotyledon*-AgNPs induced a significant increase in ROS levels in KMST-6 cells (Figure 1), while cells treated with hydrogen peroxide (the positive control) showed a significant increase in the percentage of cells with increased ROS. Similarly, in a study by Gliga et al. (2014), polyvinylpyrrolidone (PVP) and citrate coated AgNPs did not induce any significant ROS increase in non-cancerous bronchial epithelial cells (BEAS-2B) [29]. However, only a few studies have shown results similar to this; many other studies have reported AgNPs to increase intracellular ROS levels in cells including A549 [26,28] and Hep G2 cells [30].

### 3.4. Effects of C. orbiculata Extracts and Cotyledon-AgNPs on Apoptosis

**Figure 2:**
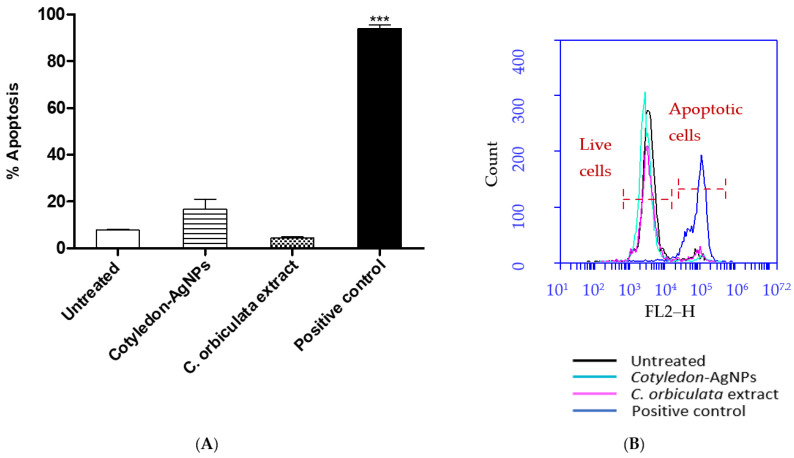
(**A**) shows the apoptotic effects of *Cotyledon*-AgNPs (2.5 µg/mL) and *C. orbiculata* extract (15 µg/mL) on KMST-6 cells. (**B**) shows an example of a histogram plot of cells stained with the APOPercentage dye and analysed for fluorescence by flow cytometry. The histogram indicates live cell populations (cells that did not take up the APOPercentage dye) and apoptotic cell populations (cells that are stained with the APOPercentage dye). Each value represents mean ± SEM (n = 3); statistical significance of the *C. orbiculata* extract- and *Cotyledon*-*AgNPs*-treated cells when compared with the untreated cells is indicated with *** *p* for <0.001.

Apoptosis is a programmed cell death process that can be induced by intracellular or extracellular signals [31]. Nanoparticles have been reported to cause mitochondrial-dependent apoptosis through increased ROS levels and membrane damage [32,33]. The loss of mitochondrial membrane potential and the impairment of membrane permeability leads to the release of proapoptotic proteins, such as cytochrome c, into the cytosol [34]. The released cytochrome c activates caspase 9, which will in-turn activate caspase 3 [35]. Caspase 3 cleaves nuclear DNA causing DNA fragmentation and eventually cell death [31]. Studies have also shown that nanoparticles induce apoptosis through activation of the P53 pathway [36]. P53 enhances the expression of proapoptotic proteins while interacting with and neutralizing the antiapoptotic proteins, therefore causing apoptosis [33,37]. The APOPercentage assay was used to determine the effects of *Cotyledon*-AgNPs and *C. orbiculata* extract on apoptosis in KMST-6 cells. As shown in Figure 2, neither the *C. orbiculata* extract or the *Cotyledon*-AgNPs had significant apoptotic effects on the cells as the levels of apoptosis in the treated cells were not significantly higher than those obtained in the untreated cells, while the wells treated with hydrogen peroxide (the positive control) showed a significantly higher percentage of apoptotic cells compared with the negative control (untreated cells).

### 3.5. Effects of Cotyledon-AgNPs on the Expression Genes Involved in Toxicity

**Figure 3:**
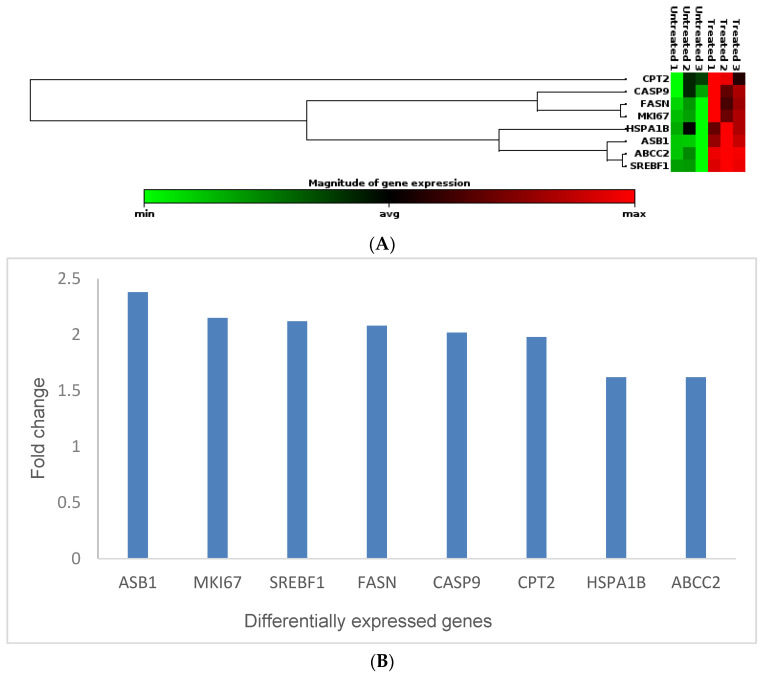
(**A**) is a clustergram and heat map of genes that are upregulated in KMST-6 cells treated with *Cotyledon*-AgNPs (2.5 µg/mL). (**B**) shows a bar chart of the fold changes of the differentially expressed genes. The fold changes, which were determined using Qiagen GeneGlobe Data Analysis Center, compare the expression levels of genes in *Cotyledon*-AgNPs-treated cells and untreated controls. The experiment was repeated 3 times.

Studying the toxicity of nanomaterials using traditional bioassays was reported to have several disadvantages, which leads to irreplicable results [38]. The nanomaterials can interfere with the bioassay, which leads to unreliable results. However, gene expression analyses are considered one of the best ways to evaluate nanomaterial toxicity [22,39]. Therefore, in this study, gene expression studies were conducted to determine the molecular effects of *Cotyledon*-AgNPs on a non-cancerous skin fibroblast cell line, KMST-6. A molecular toxicity panel (Human Molecular Toxicology PathwayFinder RT2 Profiler PCR Array, Qiagen, Hilden, Germany) consisting of 84 genes was used for this analysis. Out of the eighty-four genes, eight (*FASN*, *SREBF1*, *CPT2*, *ASB1*, *HSPA1B*, *ABCC2*, *CASP9*, and *MKI67*) were differentially expressed in KMST-6 cells treated with 2.5 µg/mL *Cotyledon*-AgNPs for 24 h. All the differentially expressed genes that were upregulated are listed in Table 2 and in Figure 3 below. Their expression levels were between 1.5 and 2.5 times higher in the *Cotyledon*-AgNPs-treated cells when compared with untreated cells.

**Figure 4:**
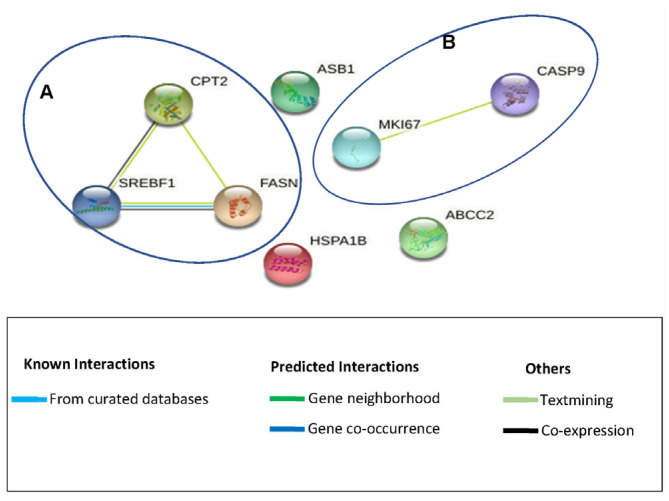
Protein networks showing the interactions between the differentially expressed genes. These networks were determined using the STRING database. Two functional network clusters (represented by A and B) were generated.

The Search Tool for the Retrieval of Interacting Genes/Proteins (STRING 11.5) was used to investigate whether these genes encode proteins that are part of functional protein–protein networks. This analysis showed that the upregulated genes clustered into two groups (denoted A and B) while some of the genes did not form part of any known functional network (Figure 4). STRING analysis demonstrated functional networks for group A between three (*FASN*, *CPT2*, and *SREBF1*) of eight genes, while another network (group B) existed for two other genes (*MKI-67* and *CASP9*). *FASN*, *CPT2*, and *SREBF1* are involved in lipid metabolism, oxidation, and haemostasis, respectively. *FASN* is involved in fatty acid metabolism; it catalyses the formation of the long-chain saturated fatty acid, palmitate, from acetyl-CoA and malonyl-CoA [40,41]. Palmitate is utilised in the production of different lipids, including phospholipids, that are used in the formation of membranes. *FASN* has mostly been associated with cancer cell proliferation; however, a study by Veigel et al. (2015) showed that *FASN* promoted the growth of normal ovarian epithelial cells [42]. Because of their findings, they described *FASN* as a marker of cell proliferation rather than a marker of cancer growth. In our previous study, it was shown that *Cotyledon*-AgNPs promote cell growth and wound healing [18]; the upregulation of *FASN* therefore supports the suggestion that the protein encoded by this gene plays a role in cell proliferation. *SERBF1*, which controls genes that are involved in lipid synthesis (e.g. *FASN*) in order to maintain cellular lipid homoeostasis [22,43,44], was also upregulated. SREBFs have been shown to connect lipid metabolism with nutrition and cell growth. In response to low cholesterol levels, SREBF1 moves from the endoplasmic reticulum to the Golgi apparatus and eventually the nucleus, where it induces the expression of genes involved in lipid synthesis [44]. *SREBF1* regulates the expression of factors required for fatty-acid synthesis, while *SREBF2* regulates those for cholesterol synthesis [43]. Because the body needs to maintain homeostasis, any action in the body has a counteraction. In this case, increased lipid production by the *Cotyledon*-AgNPs-treated cells possibly led to the upregulation of the *CPT2* gene. *CPT2* is found on the inner membrane of the mitochondria, where it is involved in fatty acid oxidation and in preserving the structure of the mitochondria [45,46]. Fatty acid oxidation is the breakdown of fatty acids into acetyl-CoA, while producing ATP and nicotinamide adenine dinucleotide phosphate (NADPH). NADPH provides the reducing power for anabolic reactions and also counteracts oxidative stress [46,47]. *CPT2* might have contributed to reduced cellular ROS levels in *Cotyledon*-AgNPs-treated KMST-6 cells (Figure 1).

The expression of *MKI-67* is used as an indicator for proliferating cells [48] as it is expressed in proliferating cells but not in resting cells. This means that at least two of the genes that are upregulated promote cell proliferation. Interestingly, in cluster B, *CASP9*, a gene that is involved in cell death, was also upregulated. *CASP9* is an apoptotic gene which is involved in the activation of the caspases responsible for apoptosis. After being activated by binding to Apaf-1, CASP9 activates other caspases including Caspase-3 and -7 [49,50]. Even though *CASP9* was upregulated, it did not induce apoptosis of the *Cotyledon*-AgNPs-treated fibroblasts, as shown by the APOPercentage assay (Figure 2) and previous studies that confirm cell growth at the concentrations used in this study [18]. It is not clear why the expression of a gene that is involved in apoptosis would be upregulated in cells that show increased proliferation. However, there is evidence that caspases that are produced in apoptotic cells can induce the proliferation of neighboring surviving cells in an effort to replace dying cells in a process referred to as “apoptosis-induced proliferation”. It is speculated that this may play a role in tissue regeneration [51].

The three genes that did not form part of any functional network are *ASB1*, *HSPA1B*, and *ABCC2*. *ASB1* is a member of the ankyrin repeat and SOCS box-containing (ASB) family and it is mainly involved in the process of ubiquitination, which includes protein modification or misfolding, which is a consequence of cell damage. *ASB1* is also linked to the expression of proinflammatory genes [52]. However, it is possible that the upregulation of *ASB1* may be countered by the upregulation of *HSPA1B*. The *HSPA1B* gene encodes a member of the heat shock protein family. Heat shock proteins (HSPs) protect cells from a range of stressors, including proteotoxic stress, by repairing misfolded or damaged proteins and thus maintaining protein function [53,54]. HSPs are critical for maintaining functional cellular pathways, protecting cell integrity, and ultimately promoting cell survival [53,54]. Hsp70 proteins have also been reported to inhibit caspase-dependent and caspase-independent apoptosis by neutralizing apoptosis-inducing factors and also inhibiting the binding of Apaf1 to procaspase-9, thus preventing its activation [55]. This may explain why no apoptotic effects were observed in the cells (Figure 2).

The third gene that was upregulated was *ABCC2*, a member of the ATP-binding cassette (ABC) family. As part of the ABC transport proteins, ABCC2 transports various compounds across the cell membranes and epithelial barriers [56,57]. ABC proteins have been reported to transport different compounds, including fatty acids and lipid compounds, across membrane barriers [58]. It is likely that ABCC2 was upregulated to transport fatty acids and lipids produced because of the upregulated *FASN*- and *SERBF1*-induced activities. The gene panel used in this study investigated the expression of several genes involved in necrosis, DNA damage, oxidative stress, endoplasmic reticulum stress, and phospholipidosis. These genes include but are not limited to *CYLD*, *GRB2* (necrosis), *BRCA1*, *MDM2* (DNA damage), *AKR1C2*, *FHL2* (oxidative Stress), *ADM2*, *ASNS* (endoplasmic reticulum stress) *ASAH1*, and *HPN* (phospholipidosis). This study shows that the expression of these genes was not affected by treatment with *Cotyledon*-AgNPs. This is in agreement with the findings of the bioassays (ROS and APOPercentage) that demonstrated that the nanoparticles are not toxic to skin fibroblasts. In fact, this gene expression study supports previous findings [18] which suggest that *Cotyledon*-AgNPs may promote cell growth as shown by the upregulation of *FASN*, *SERBF1*, *MKI-67*, and *HSPA1B*.

## 4. Conclusions

It can be concluded from the various bioassays used in this study that the *C. orbiculata* extract and *Cotyledon-*AgNPs are not toxic to KMST-6 cells at 15 and 2.5 µg/mL concentrations, respectively. Treatments with the *C. orbiculata* extract or *Cotyledon-*AgNPs did not induce oxidative stress or apoptosis in these cells. This finding is supported by gene expression analysis which shows that the expression of genes involved in toxicity was largely not affected in KMST-6 cells subjected to *Cotyledon-*AgNPs treatment. Gene expression studies mainly showed the upregulation of genes involved in fatty acid metabolism and mitochondrial energy metabolism. The upregulation of genes involved in lipid metabolism (*FASN* and *SERBF*1) and cell proliferation (*MKI-67*) also support previous findings that *Cotyledon-*AgNPs can promote wound healing by increasing the growth rate of cells involved in would healing, such as skin fibroblast cells. Due to the ability of *Cotyledon-*AgNPs to promote the proliferation and migration of cells involved in wound healing, its low cytotoxicity towards these cells, and its high antimicrobial activity towards microbes that are known to infect wounds, *Cotyledon-*AgNPs can potentially be used as highly effective wound-healing agents. However, this assumption is entirely based on results we obtained using in vitro studies. The translational gap between in vitro studies and in vivo studies is well documented. Therefore, extensive research still needs to be undertaken using animal models to validate the bioactivities of *Cotyledon*-AgNPs.

## Figures and Tables

**Table 1:** IC_50_ values of *C. orbiculata* extract and the *Cotyledon*-AgNPs in cell cultures.

		IC_50_ (µg/mL)	
Treatment	KMST-6	HaCaT	CHO
*C. orbiculata* extract	296 ± 27.34	>1000	>1000
*Cotyledon*-AgNPs	122 ± 19.30	40.55 ± 2.68	21.08 ± 0.76

**Table 2:** Cytotoxicity genes upregulated in *Cotyledon*-AgNPs-treated cells.

Gene Name	Symbol	Function
Fatty acid synthase	FASN	Steatosis
Sterol regulatory element-binding transcription factor 1	SREBF1	Steatosis
Carnitine palmitoyl transferase II	CPT2	Fatty Acid Metabolism (β-Oxidation)
Ankyrin repeat and SOCS box protein 1	ASB1	Mitochondrial Energy Metabolism
Heat Shock Protein Family A (Hsp70) Member 1B	HSPA1B	Mitochondrial Energy Metabolism
ATP Binding Cassette Subfamily C Member 2	ABCC2	Cholestasis
Caspase-9	CASP9	Apoptosis
Marker Of Proliferation Ki-67	MKI67	Immunotoxicity

## Data Availability

Supporting data presented in this study are available on request from the corresponding author.
